# No evidence of a causal relationship between ankylosing spondylitis and cardiovascular disease: a two-sample Mendelian randomization study

**DOI:** 10.3389/fcvm.2023.1243867

**Published:** 2023-10-12

**Authors:** Yan Zhong, YingWen Chen, XinYue Zhang, WenJun Cai, ChangWei Zhao, WenHai Zhao

**Affiliations:** ^1^ College of Traditional Chinese Medicine, Changchun University of Chinese Medicine, Changchun, China; ^2^ College of Traditional Chinese Medicine, Tianjin University of Chinese Medicine, Tianjin, China; ^3^ College of Integrated Chinese and Western Medicine, Changchun University of Chinese Medicine, Changchun, China; ^4^ Department of Orthopedics, The Third Affiliated Clinical Hospital of Changchun University of Chinese Medicine, Changchun, China; ^5^ Department of Orthopedics, Affiliated Hospital of Changchun University of Chinese Medicine, Changchun, China

**Keywords:** epidemiology, Mendelian randomization, cardiovascular disease, ankylosing spondylitis, genome-wide association study

## Abstract

**Objective:**

Observational studies have suggested an increased risk of cardiovascular disease in individuals with ankylosing spondylitis. However, these studies are prone to confounding factors and reverse causality. To address these limitations, we conducted a Mendelian randomization study to assess the causal relationship between AS and CVD.

**Methods:**

The study population comprises 9,069 individuals with ankylosing spondylitis and 509,093 individuals with either of six common cardiovascular diseases and a related indicator. Causal analysis using summary effect estimates and inverse variance weighting were employed as the main methods.

**Results:**

The CAUSE analysis showed no evidence of a causal relationship between AS and CVD. The odds ratios for total CVD, heart failure, myocardial infarction, valvular heart disease, ischemic heart disease, and venous thromboembolism, Arterial stiffness index, were as follows: OR, 1.01; 95% confidence interval, 0.96–1.05; *P* = 0.91; OR, 1.03; 95% CI, 0.99–1.08; *P* = 0.50; OR, 0.94; 95% CI, 0.86–1.03; *P* = 0.53; OR, 0.99; 95% CI, 0.94–1.04; *P* = 0.99; OR, 0.98; 95% CI, 0.91–1.04; *P* = 0.94; OR, 0.98; 95% CI, 0.91–1.04; *P* = 0.99; β, −0.0019; 95% CI, 0.97–1.01; *P* = 0.99. The IVW and weighted median methods also yielded consistent results, and no heterogeneity or pleiotropy was found. Likewise, a reverse Mendelian randomization analysis did not uncover a heritable causal relationship between AS and CVD.

**Conclusion:**

This Mendelian randomization study does not support a causal relationship between AS and CVD. Further research is needed to confirm this association.

## 1. Introduction

Cardiovascular disease (CVD) is a condition that impacts the heart and blood vessels, and it stands out as having the highest mortality and morbidity rates among non-infectious diseases (1). Over the past decade, there has been a notable 12.5% increase in CVD-related deaths, primarily attributed to the aging population (2). The escalating burden of CVD presents a significant challenge to both society and families, underscoring the crucial need to identify risk factors associated with CVD and implement effective preventive measures.

Ankylosing Spondylitis (AS) is a chronic inflammatory disease classified as a form of axial spondyloarthritis (3). In the United States, it affects approximately 0.9% to 1.4% of adults, which is comparable to the prevalence of rheumatoid arthritis (4). AS primarily manifests as chronic inflammatory low back pain, peripheral arthritis, and sacroiliac arthritis (5). Patients with AS commonly experience restricted spinal mobility as a result of back pain and stiffness, and over time, they may develop progressive hyperkyphosis (6), leading to compromised cardiopulmonary function (7).

Observational studies have demonstrated an association between AS and increased morbidity and mortality related to CVD (8, 9). However, the causal relationship between AS and CVD remains contentious. Cohort studies have reported an elevated risk of ischemic heart disease (IHD) and myocardial infarction (MI) in AS patients, yet multiple studies have failed to reproduce these findings (10). Furthermore, there is ongoing controversy surrounding the association between AS and atherosclerosis (11, 12). It is worth noting that comorbidities commonly observed in AS, such as hypertension (HTN) and diabetes mellitus (DM) (13), along with well-established risk factors like smoking, obesity, and reduced physical activity, are strongly associated with CVD morbidity and mortality (14, 15). Consequently, it remains uncertain whether the development of CVD is directly attributed to AS itself or indirectly influenced by the comorbidities associated with AS. This uncertainty arises from inherent limitations in previous studies, including small sample sizes, issues of reverse causality, and potential confounding factors.

Mendelian randomization (MR) is a statistical method that uses genetic variation as an instrumental variable (IV) to assess causal relationships between exposures and outcomes, particularly in situations where randomized controlled trials (RCTs) are not feasible or practical (16). One of the key advantages of MR is its ability to overcome issues of confounding, as genetic variants are typically not influenced by environmental factors or disease status (17). Another advantage of MR is the potential for larger sample sizes. This is particularly useful when traditional observational studies may not have enough statistical power to detect a causal relationship (18). Therefore, our goal is to explore the causal relationship between AS and CVD through two sample MR analysis.

## 2. Methods and materials

### 2.1. Study design

**Figure 1:**
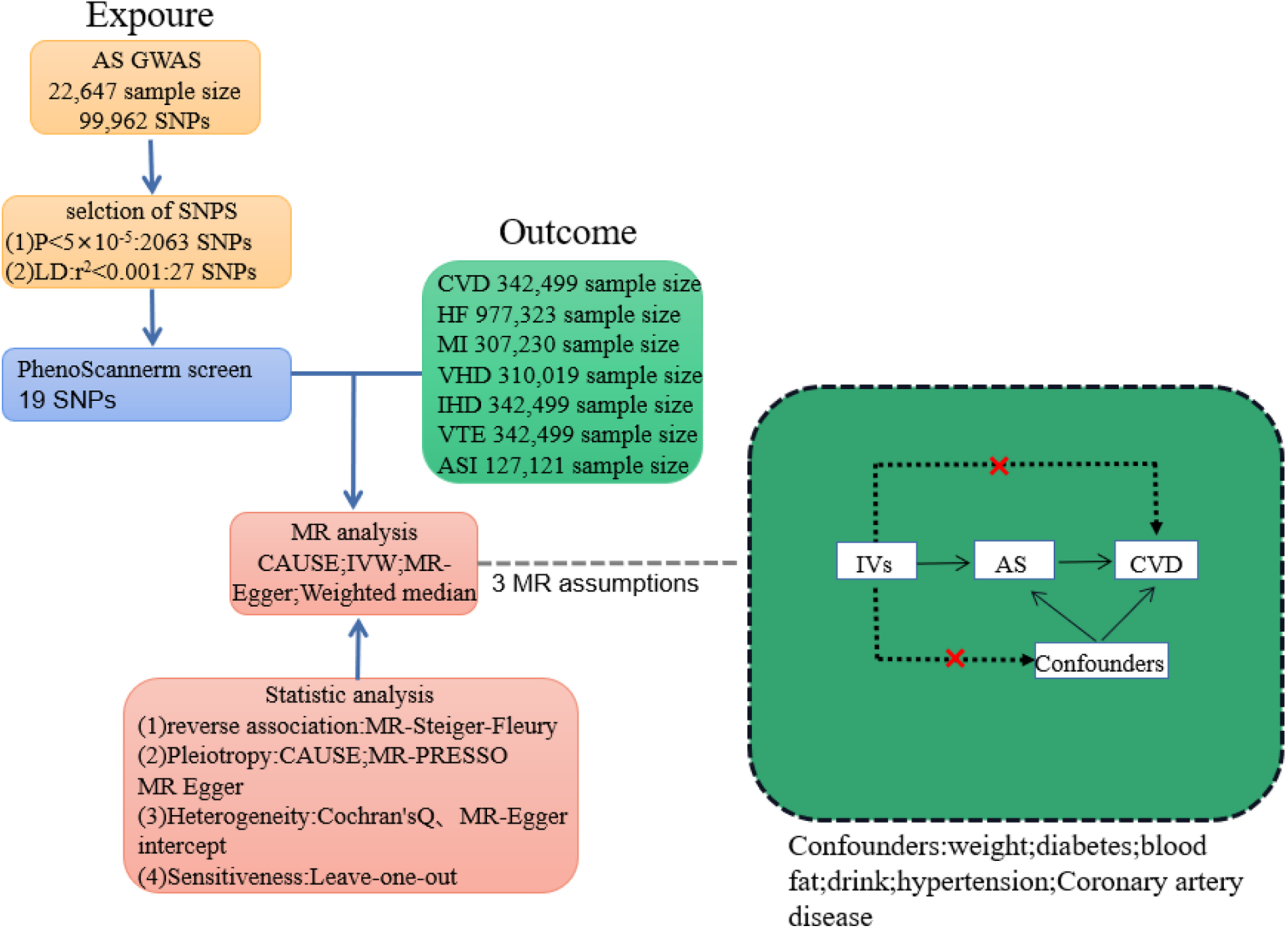
Plot of Mendelian randomization study between ankylosing spondylitis and cardiovascular disease. IVs, instrumental variable; CVD, total cardiovascular disease; HF, heart failure; MI, myocardial infarction; VHD, heart valve disease; CHD, coronary heart disease; IHD; ischaemic heart disease; VTE, venous embolism; ASI, arterial stiffness index.

In MR analysis, genetic variations associated with the exposure are employed as instrumental variables (IVs) to explore the causation between exposure and outcome. The design of a Mendelian randomization study must adhere to three fundamental assumptions: First, the instrumental variables and exposure should exhibit a strong association. Second, the instrumental variables should be independent of confounding factors. And third, the instrumental variables should solely impact the outcome through their influence on the exposure (Figure 1).

### 2.2. Instrumental variable selection

Instrumental variables for AS were used from the GWAS genome-wide data of Crotes et al. (19). The total sample size was 22,647 Europeans (9,069 ncase and 13,578 ncontrol). To fulfill the first hypothesis of the MR analysis, we employed three selection methods. Firstly, SNPs with a significance threshold of *P* < 5 × 10^−8^ were chosen. Secondly, any screened genes displaying linkage disequilibrium (*R*^2 ^< 0.001; distance <10,000 kb) were excluded to ensure the independence of genetic variations. Thirdly, to prevent weak instrumental variables from biasing our findings, we used the F-value statistic (F = beta^2^/se^2^). If the calculated F-statistic was greater than 10, we concluded that the relationship between IV and exposure was sufficiently strong and would not affect the results of our MR analysis (20). In cases where the requested SNPs were unavailable, we supplemented them using SNPs from the 1,000 Genomes Project proxy (LD; *r*^2 ^> 0.8) (21).

To mitigate potential confounding effects, we consulted the PhenoScanner database (http://www.phenoscanner.medschl.cam.ac.uk/) to assess whether the selected SNPs violated hypotheses (2) and (3). As a result, six traits (i.e., weight, cardiovascular disease, alcohol, blood glucose, total cholesterol and hypertension) were found to be potential confounders. Thus, we further excluded SNPs strongly associated with these six potential confounders as well as CVD to ensure that the resulting instrumental variables influenced CVD only through AS, with no confounding factors affecting the final results (22). After examining the potential synergistic effects between exposure and the outcome, we excluded SNPs with palindromic properties. Ultimately, we identified 19 SNPs as genetic variables for the instrumental variable (IV) analysis (Table 1).

**Table 1:** As instrumental variables.

SNP	Other	Effect	*P* value	Beta	SE	Potential confounding factors	*F* value
rs6600247	C	T	2.58×10^−15^	−0.032833157	0.004150884		62.57
rs11209026	G	A	1.94×10^−27^	−0.103584316	0.009544807		117.78
rs4129267	G	A	3.32×10^−13^	−0.030768534	0.00422605	Coronary artery disease	
rs1801274	G	A	1.35×10^−09^	−0.025317808	0.004176873		35.89
rs41299637	T	G	1.81×10^−15^	−0.039052727	0.00491011		26.59
rs1250550	C	A	1.46×10^−9^	−0.026036015	0.00430465		36.58
rs11190133	C	T	4.84×10^−14^	−0.03386707	0.004494013		56.79
rs1860545	C	T	2.78×10^−10^	−0.027473977	0.004353781		39.82
rs11065898	G	A	4.71×10^−8^	0.026252371	0.004806405	High blood pressure	
rs11624293	T	C	1.49×10^−10^	0.0428679	0.006691612		41.04
rs75301646		A	2.58×10^−9^	0.02508278	0.004211266	Alcohol	
rs2531875	A	C	1.22×10^−10^	0.027323296	0.004244632		41.44
rs9901869	T	C	6.04×10^−15^	−0.031903549	0.004088526	Total cholesterol	
rs35164067	G	A	3.43×10^−10^	−0.031077956	0.004950207		39.41
rs6759298	G	C	4.81×10^−47^	0.059588791	0.004136652		207.51
rs12615545	T	C	1.03×10^−9^	0.025472796	0.004172729		37.27
rs4676410	G	A	9.90×10^−9^	0.02810143	0.004902145		32.86
rs2836883	G	A	6.46×10^−17^	−0.039676817	0.004747979		69.83
rs743479	A	G	2.03×10^−8^	0.023423515	0.004175808		31.46
rs27529	G	A	3.28×10^−47^	0.062035391	0.004298593	Weight	
rs6556416	G	T	4.22×10^−8^	−0.025215249	0.0046002		30.05
rs9261288	C	T	5.92×10^−10^	0.064944689	0.010487486		38.35
rs115893523	T	A	6.64×10^−43^	0.195649017	0.014248878		188.56
rs10456384	G	C	1.35×10^−161^	0.224742273	0.008296614		733.78
rs2240804	G	A	2.24×10^−118^	0.098215047	0.004245972	Weight	
rs1576	T	C	1.00×10^−200^	−0.14618251	0.004811583	Diabetes	
rs1128905	C	T	6.95×10^−9^	0.023716527	0.004094588	Fasting glucose	

### 2.3. Sources of data for outcome

Data for total cardio-vascular disease (CVD) (*n* = 174,499 cases, *n* = 168,000 controls), myocardial infarction (MI) (*n* = 21,609 cases, *n* = 285,621 controls), valvular heart disease (VHD) (*n* = 72,756 cases, *n* = 237,263 controls), ischemic heart disease (IHD) (*n* = 56,730 cases, *n* = 285,769 controls), and venous thromboembolism (VTE) (*n* = 17,048 cases, *n* = 325,451 controls) were extracted from the Finnish database. The FinnGen research project aggregated the final genetic data for various diseases from the Finnish Biobank and the Finnish Health Registry (23). Arterial stiffness, which offers a different perspective on hemodynamic alterations (24), is closely associated with the development of CVD (25). Therefore, we retrieved summary-level data on the arterial stiffness index (ASI) for 127,121 participants from the UKB database (26). The data were obtained by measuring finger pulse waveforms of the participants (25). Statistics regarding heart failure (HF) were sourced from the Heart Failure Molecular Therapeutic Target Epidemiology (HERMES) Consortium, which includes 47,309 cases and 930,014 individuals of European ancestry (27).

The data used in this study were obtained from publicly available GWAS databases that previously obtained ethical approval, eliminating the need for further ethical approval. To minimize data bias caused by ethnic differences, the study population was limited to individuals of European descent, and information on the exposure and outcomes is presented in (Table 2).

**Table 2:** Mendelian randomization analysis data details.

Trait	GWAS-ID	PMID	Population	Numbers of SNPs	Sample size
Ankylosing spondylitis	ebi-a-GCST005529	23749187	European	99,962	ncase:9069
					ncontrol:13,578
Cardiovascular diseases	finn-b-FG_CVD	NA	European	16,380,466	ncase:174,499
					ncontrol:168,000
Myocardial infarction	finn-b-I9_MI_STRICT	NA	European	16,380,466	ncase:21,609
					ncontrol:285,621
Valvular heart disease	finn-b-I9_VHD	NA	European	20,168,500	ncase:72,756
					ncontrol:237,263
Ischemic heart disease	finn-b-I9_ISCHHEART	NA	European	16,380,466	ncase:56,730
					ncontrol:285,769
Arterial stiffness index	GCST008403	31235810	European	13,295,130	ncase:127,121
Venous thromboembolism	finn-b-I9_VTE	NA	European	16,380,466	ncase:17,048
					ncontrol:325,451
Heart failure	HERMES	31919418	European	8,281,262	ncase:47,309
					ncontrol:930,014

### 2.4. Statistical analyses

In this study, we used the “TwoSampleMR”, “MRPRESSO”, and “CAUSE” packages in R (4.2.3) for data analysis. The study adhered to the STROBE-MR statement (28). To address the potential impact of reverse association (i.e., CVD for exposure and AS for outcome), we applied the MR-Steiger-Fleury method and reverse MR methods. These two methods helped us ensure that all IVs were correctly oriented in our inferences. We assessed the directionality of the association between them and employed bidirectional MR to investigate their potential causal relationship.

We employed the Causal Analysis Using Summary Effect estimates (CASUSE) approach for causal analysis (29). The issue of horizontal pleiotropy has been a challenge in MR studies, wherein it can be categorized as “correlated horizontal pleiotropy” and “uncorrelated horizontal pleiotropy” (30). While previous studies have addressed the impact of “uncorrelated horizontal pleiotropy” using MR-PRESSO (31), ignoring the effect of “correlated horizontal pleiotropy” can result in a high false-positive rate in MR studies (32). Thus, we used CAUSE, which is superior to other established methods in detecting causality in the presence of pleiotropy (29). CAUSE leverages genome-wide summary statistics that distinguish causality from correlated pleiotropy. Additionally, Bayesian modeling is employed to further evaluate uncorrelated horizontal pleiotropy to ensure the rigor of the final conclusions.

The primary method for detecting causality in MR analyses is inverse variance weighting (IVW), provided there is no multiplicity of effects and all instrumental variables are valid (33). We used weighted median (WM) analysis as a complementary measure (34). For identifying fixed-point pleiotropy, we employed MR-Egger and used MR-PRESSO analysis for detecting unrelated pleiotropy (31). SNPs with pleiotropy were excluded when it occurred. We assessed SNP homogeneity using both the Cochran Q test and MR-Egger intercept test (35). Sensitivity analyses via the “leave-one-out” methods were conducted to investigate the effects of individual SNPs on MR.

## 3. Results

The F-value statistics for selected IVs in MR analysis ranged from 26 to 733, showing a strong prediction of AS by IVs and validating the MR hypothesis (1). The MR-Steiger-Fleury test did not reveal the presence of reverse causality (Table 3). Reverse MR analysis also did not reveal a causal relationship between CVD and AS (Supplementary Tables S1, S2).

**Table 3:** A directional test for Mendelian randomization about the effect of AS on CVD.

Outcome	SNP *r*^2^ exposure	SNP *r*^2^ outcome	Correct causal direction	Steiger *p*-value
Cardiovascular diseases	0.2714127	0.0002	TRUE	0
Heart failure	0.2743676	0.0703	TRUE	3.181036e-2210
Myocardial infarction	0.2714127	0.0002	TRUE	
Valvular heart disease	0.2714127	8166013e-05	TRUE	0
Ischemic heart disease	0.2714127	0.0002	TRUE	0
Venous thromboembolism	0.2714127	0.0001	TRUE	0
Arterial stiffness index	0.2416084	0.0002	TRUE	0

SNP r^2^ exposure: r-squared values of the exposed SNP; SNP *r*^2^ outcome: *r*-squared values of the outcome SNP; correct causal direction: AS to CVD directional correctness; Steiger *p*-value: the *p*-value of the MR-Steiger-Fleury test.

**Figure 2:**
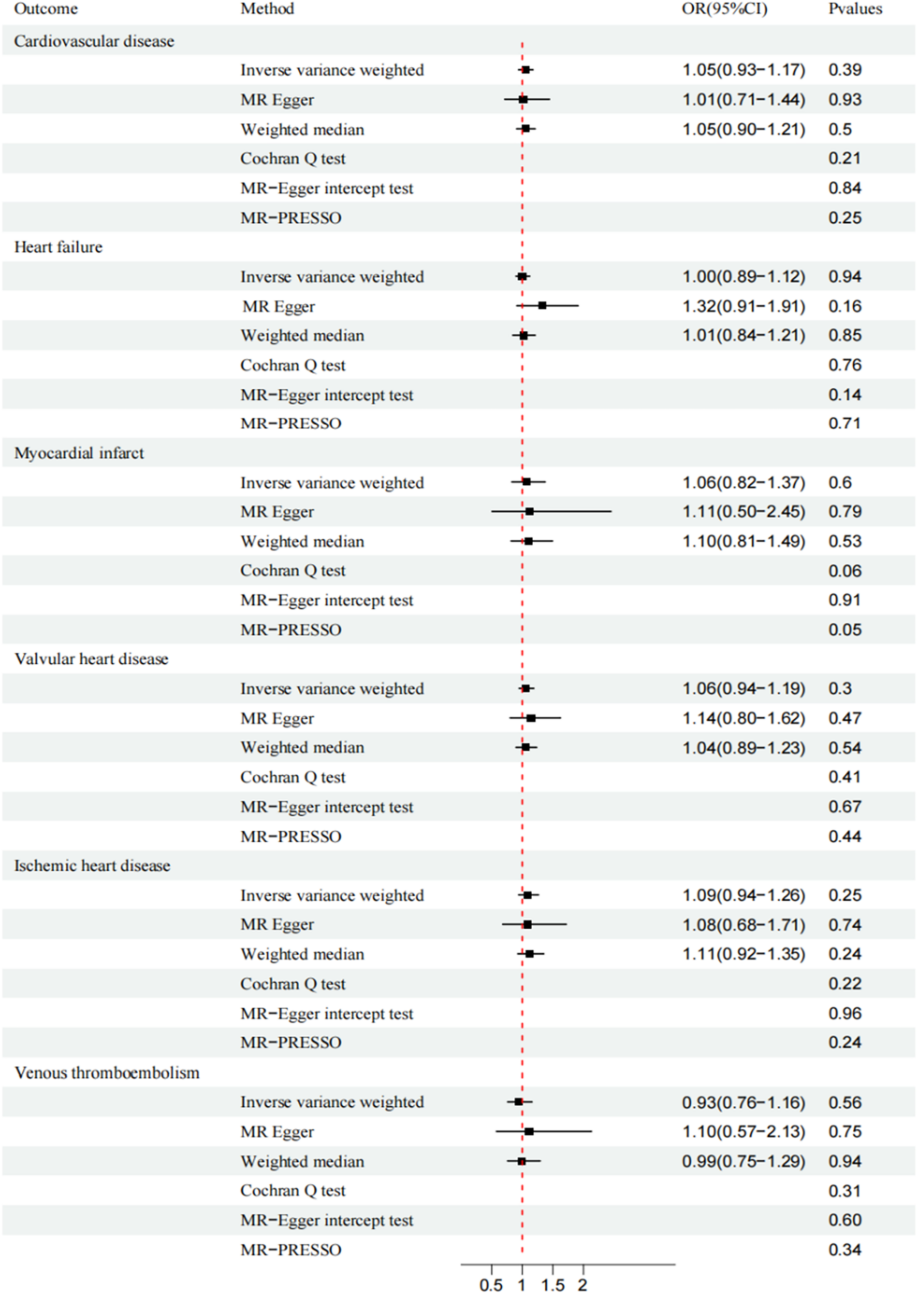
Mendelian randomization analysis of ankylosing spondylitis and cardiovascular disease. CI, confidence interval; OR, odds ratio.

**Figure 3:**
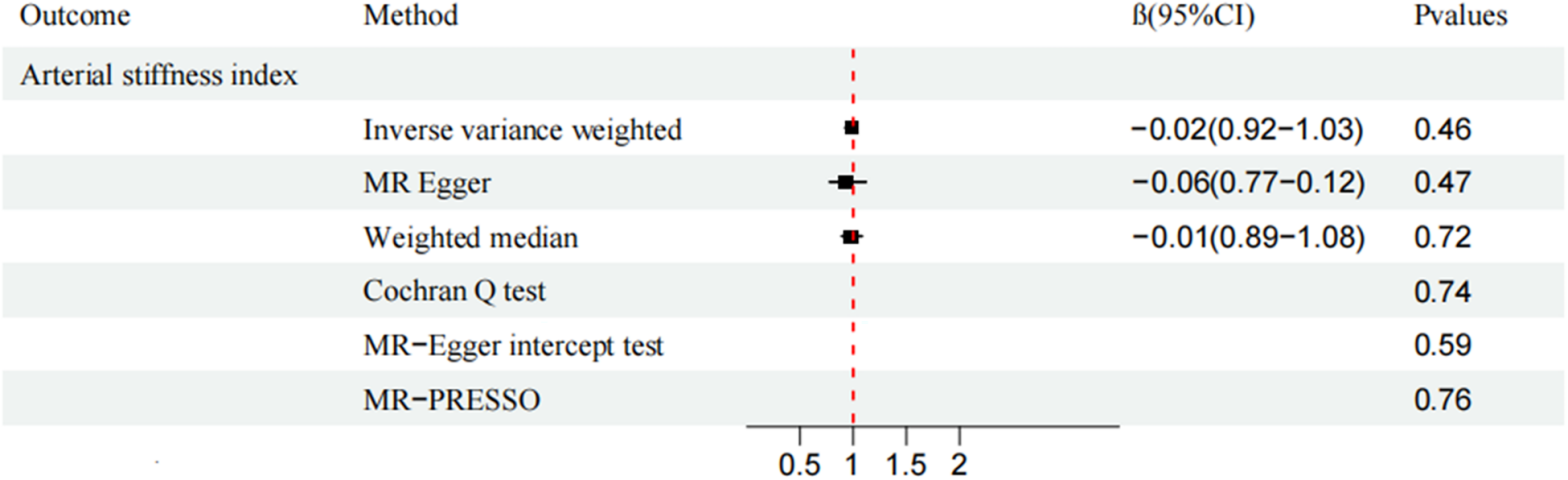
Mendelian randomization analysis of ankylosing spondylitis and arterial stiffness index.

CAUSE analysis revealed no association between AS and CVD (total CVD: OR, 1.01; 95% CI, 0.96–1.05; *P* = 0.91; HF: OR, 1.03; 95% CI, 0.99–1.08; *P* = 0.50; MI: OR, 0.94; 95% CI, 0.86–1.03; *P* = 0.53; VHD: OR, 0.99; 95% CI, 0.94–1.04; *P* = 0.99; IHD: OR, 0.98; 95% CI, 0.91–1.04; *P* = 0.94; VTE: OR, 0.98; 95% CI, 0.91-1.04; *P* = 0.99; ASI: β, −0.0019; 95% CI, 0.97–1.01; *P* = 0.99;). Similarly, the trends of IVW and WM were consistent (Figures 2, 3).

MR-Egger and MR-PRESSO secondary analyses found no fixed-point or horizontal pleiotropy in the MR analysis, and both analyses revealed identical trends. The Cochran Q test and MR-Egger intercept test indicated low or no heterogeneity (Figures 2, 3). The leave-one-out method found no evidence of overall instability or sensitivity abnormalities when eliminating one SNP. The absence of potentially confounding SNPs, the low or no heterogeneity, and the results of the multipotency robustness analysis confirmed the validity of MR hypotheses (2) and (3). For more detailed information, please refer to (Supplementary Table S3).

## 4. Discussion

In our current study, we explored the causal relationship between AS and CVD using multiple large open databases. The MR analyses conducted did not demonstrate any effect of AS on CVD, with consistent outcomes across all analyses. The findings contradicted several observational studies that have implied a causal connection between AS and CVD. As the evidence for the causal relationship between AS and CVD remains inconclusive (36), the results of our MR analysis are of great interest.

Atherosclerosis is well known as the basis of cardiovascular disease (37). Research has demonstrated differences in extracardiac pulse wave velocity (PWV) between individuals with AS and control groups (38), and two observational studies have corroborated that AS patients are at higher risk for atherosclerosis (39, 40). Two recent meta-analyses have also suggested that AS patients may have an increased of developing atherosclerosis (41, 42). However, some disagreement persists around this issue; studies have found that atherosclerosis does not accelerate when controlling for inflammatory manifestations and disease progression in AS patients (43, 44). Evidence also indicates that individuals with AS who have a shorter disease duration do not exhibit increased carotid or femoral artery plaque thickness (43). It should be noted that although observational studies have made efforts to avoid confounding factors, issues such as selection bias and reverse causality inherent in design have the potential to skew results. As a result, the causal relationship between AS and atherosclerosis remains inconclusive.

The heightened risk of CVD in individuals with AS may be attributed to the significant overlap between conventional CVD risk factors and the comorbidities of AS, such as hypertension, dyslipidemia, diabetes, smoking, and obesity (45). This is corroborated by a recent meta-analysis which reported hypertension and hyperlipidemia to be the most prevalent comorbidities among AS patients (46). Findings from another cross-sectional study also indicated that AS patients have a higher prevalence of diabetes and smoking compared to the general population (47), and that obesity is a notable issue due to various reasons (48). Being overweight or obese can have a direct impact on AS activity (49), with evidence suggesting that it can lead to reduced efficacy of biologic medications (50), and an increased likelihood of exercise-induced hypertensive (51). Matters are further complicated by the role of smoking, and in 2,021, the European League Against Rheumatism (EULAR) issued guidelines for managing cardiovascular risks, which includes cigarette cessation as a key strategy (52). The reason is that, in addition to increasing the risk of CVD (53), smoking also undermines the effectiveness of drug therapy (54). Therefore, smoking assumes a dual role in individuals with AS: on one hand, it embodies a traditional CVD risk factor; on the other hand, it influences and worsens disability and the development of AS, which ultimately leads to a more sedentary lifestyle—an additional conventional CVD risk factor (55, 56).

AS triggers a systemic inflammatory response that plays a significant role in the development of CVD (57). Cardiovascular inflammation is closely associated with elevated inflammatory cytokines, particularly interleukin-6 (IL-6) (58, 59). In patients with AS, total cholesterol and high-density lipoprotein (HDL) levels are lower than in the general population, which is closely linked to their own inflammatory activity (60). When IL-6 is increased, total cholesterol and HDL levels decrease significantly (61). IL-6 also interacts with the hypothalamic-pituitary-adrenal axis, resulting in increased insulin sensitivity that triggers diabetes, as well as increased BMI and blood pressure that lead to hypertension (62). Non-steroidal anti-inflammatory drugs (NSAIDs) are recommended as a first-line treatment to alleviate inflammation and pain (63). However, although NSAIDs have good anti-inflammatory and analgesic effects, their cardiovascular side effects are a concern for physicians, and the US Food and Drug Administration (FDA) has recently issued a warning (64). A meta-analysis found that almost all NSAIDs increase the risk of MI (65). Even short-term NSAID use can lead to an increase in thrombotic cardiovascular events (66). Real-world studies conducted in four European countries discovered that NSAIDs increased the risk of heart failure death in patients (67), and high doses of diclofenac or ibuprofen can more than double this risk. The administration of NSAIDs can also lead to adverse effects on thrombosis. Several studies suggest that even short-term concurrent use of NSAIDs and antithrombotics increases the risk of bleeding, with no specific window period (68, 69).

Observational studies have indicated a potential association between ankylosing spondylitis and the development of VHD (70). But our MR analysis did not reveal any evidence supporting a genetic causative link between ankylosing spondylitis and VHD. Nevertheless, patients with AS frequently exhibit clinical manifestations linked to VHD, such as aortic valve insufficiency and aortic inflammation (71). Notably, individuals positive for the HLA-B27 marker often exhibit an increased aortic root index, which correlates with a heightened risk of complications like aortic valve regurgitation (AVR) (72). In the real-world scenario, HLA-B27 positivity is associated with sustained activation of IL-23 + T cells, leading to an inflammatory response within the heart (73). A comprehensive cohort study involving AS patients from an East Asian population suggested that chronic systemic inflammation may contribute to the early development of VHD (74). Furthermore, a related study has solidified the connections among aortic stiffness, myocardial function, and the activity of AS (75). These findings align with autopsy results, which reveal aortic valve fibrosis in AS patients (76). However, the relationship between AS and cardiovascular disease is profoundly intricate. Given the existence of different AS subtypes with varying manifestations across diverse racial and gender groups, it is imperative to conduct more extensive research to thoroughly explore the associations between different AS subtypes, races, and CVD (77).

Recent genetic and MR studies have demonstrated the role of IL-6 in atherosclerosis and CVD (78, 79). Although our MR analysis did not reveal a causal link between AS and CVD, we identified the IL-6R SNP rs4129267 as a pleiotropic SNP that was removed from our analysis. Given the critical role of IL-6 in CVD, we postulated that specific loci such as IL-6 or local genomic correlations might be responsible for the relationship between AS and CVD. Further analysis using a co-localization approach would help elucidate this relationship. However, we must note that the usage of rs4129267 as a potential therapeutic target for CVD would require in-depth demonstration via clinical studies.

## 5. Conclusion

Our study provides a unique perspective on the prevention and treatment of CVD in patients with AS. Although our MR analysis does not find a genetic causal relationship between AS and CVD, we acknowledge the need for caution while interpreting our findings. Given the comparatively lower appreciation for the prevention and management of CVD comorbidities in patients with AS, we believe additional experimental studies and clinical research are necessary to inform evidence-based interventions.

### 5.1. Advantages

Our study has several strengths. Firstly, the utilization of the MR approach facilitated the avoidance of interference from environmental factors, inherent bias, and reverse causality into the study's outcomes. Secondly, through a rigorous screening process, we minimized confounding factors that could affect the final results. Lastly, we employed multiple analytical techniques such as CAUSE, IVW, and WM to ensure the rigorousness of the study's findings regarding the causal relationship between AS and CVD.

### 5.2. Limitations

Our study has several limitations. Firstly, our population was restricted to European populations, which could limit the generalization of the findings to other races. Secondly, AS is a progressive disease with different comorbidities at each stage, which could influence the relationship between AS and CVD in various ways. Although we made efforts to include as many cardiovascular diseases associated with AS as possible, some cardiovascular events that were not considered in our study may also influence the relationship between AS and CVDs. Thirdly, we have five GWAS datasets derived from the FinnGen database which introduces certain limitations and the possibility of selection bias. In future studies we will incorporate more diverse data for more in-depth research. Further studies that address these limitations may provide a more comprehensive understanding of the relationship between AS and CVDs.

## Data Availability

The original contributions presented in the study are included in the article/**Supplementary Material**, further inquiries can be directed to the corresponding author/s.
