# Supplementary material for: No evidence of a causal relationship between ankylosing spondylitis and cardiovascular disease: a two-sample Mendelian randomization study
Source: Front Cardiovasc Med. 2023 Oct 12;10:1243867. doi: 10.3389/fcvm.2023.1243867 (PMC10600491; doi:10.3389/fcvm.2023.1243867)

Supplementary Material

No Evidence of a Causal Relationship between Ankylosing Spondylitis and Cardiovascular Disease: A Two-Sample Mendelian Randomization Study

Yan Zhong^1†^, Ying Wen Chen^2†^, Xin Yue Zhang^3^, Wen Jun Cai^4^, Chang Wei Zhao^5*^, Wen Hai Zhao^5*^

*** Correspondence:** Corresponding Author: [zhaocw@ccucm.edu.cn](mailto:zhaocw@ccucm.edu.cn);zwh9899@163.com

# Supplementary Data

**Supplementary Table 1**. MR analysis with exposure to AS as the outcome in CVD

| Exposure | Method | nsnps | P value | OR (95%CI) |
| --- | --- | --- | --- | --- |
| Cardiovascular diseases | Inverse variance weighted | 2 | 0.94785 | 0.99（0.85-1.17） |
| Heart failure | Wald ratio | 1 | 0.786169 | 1.02（0.88-1.89） |
| Ischemic heart disease | Inverse variance weighted | 3 | 0.908279 | 1.01（0.88-1.56） |
| Ischemic heart disease | MR Egger | 3 | 0.476831 | 1.45（0.89-1.48） |
| Ischemic heart disease | Weighted median | 3 | 0.638988 | 1.03（1.03-1.17） |
| Myocardial infarction | Inverse variance weighted | 3 | 0.917508 | 0.00（0.94-1.06） |
| Myocardial infarction | MR Egger | 3 | 0.698138 | 0.97（0.86-1.09） |
| Myocardial infarction | Weighted median | 3 | 0.89715 | 1.00（0.95-1.06） |
| Venous thromboembolism | Wald ratio | 1 | 0.307079 | 0.98（0.95-1.02） |
| Arterial stiffness index | Inverse variance weighted | 0 | NA | NA |
| Arterial stiffness index | MR Egger | 0 | NA | NA |
| Arterial stiffness index | Weighted median | 0 | NA | NA |
| Valvular heart disease | Wald ratio | 1 | 0.403586 | 0.94（0.82-1.08） |

**Supplementary Table 2.** Heterogeneity sensitivity and pleiotropic testing for exposure to AS as outcome in CVD

| Exposure | Cochran Q test | | MR-Egger intercept test | | MR-PRESSO |
| --- | --- | --- | --- | --- | --- |
|  | Q value | P Q | Intercept | P | P value |
| Cardiovascular diseases | 1.24 | 0.27 | NA | NA | NA |
| Heart failure | 2.02 | 0.96 | NA | NA | NA |
| Myocardial infarction | 3.17 | 0.21 | 0.006 | 0.60 | NA |
| Valvular heart disease | NA | NA | NA | NA | NA |
| Ischemic heart disease | 3.67 | 0.16 | -0.009 | NA | NA |
| Venous thromboembolism | NA | NA | NA | NA | NA |
| Arterial stiffness index | NA | NA | NA | NA | NA |

**Supplementary Table 3** Heterogeneity sensitivity and pleiotropy testing for AS exposure to CVD as outcome heterogeneity

| Outcome | Cochran Q test | | MR-Egger intercept test | | MR-PRESSO |
| --- | --- | --- | --- | --- | --- |
|  | Q value | P Q | Intercept | P | P value |
| Cardiovascular diseases | 17.83 | 0.21 | 0.0012 | 0.84 | 0.25 |
| Heart failure | 10.86 | 0.76 | -0.0099 | 0.14 | 0.71 |
| Myocardial infarction | 22.76 | 0.06 | -0.0014 | 0.91 | 0.05 |
| Valvular heart disease | 14.51 | 0.41 | -0.0025 | 0.67 | 0.44 |
| Ischemic heart disease | 17.70 | 0.22 | 0.0003 | 0.96 | 0.24 |
| Venous thromboembolism | 15.92 | 0.31 | -0.0059 | 0.60 | 0.34 |
| Arterial stiffness index | 10.23 | 0.74 | 0.0017 | 0.59 | 0.76 |

Table 3. Ankylosing spondylitis and CVD heterogeneity and horizontal multiplicity tests: Q value: statistic of Cochrane Q test; PQ: p-value of Cochrane's Q test; Intercept: egger intercept; P: p-value of MR-Egger intercept test; P value: MR-PRESSO Global Test p-value.

## Supplementary Figures

**
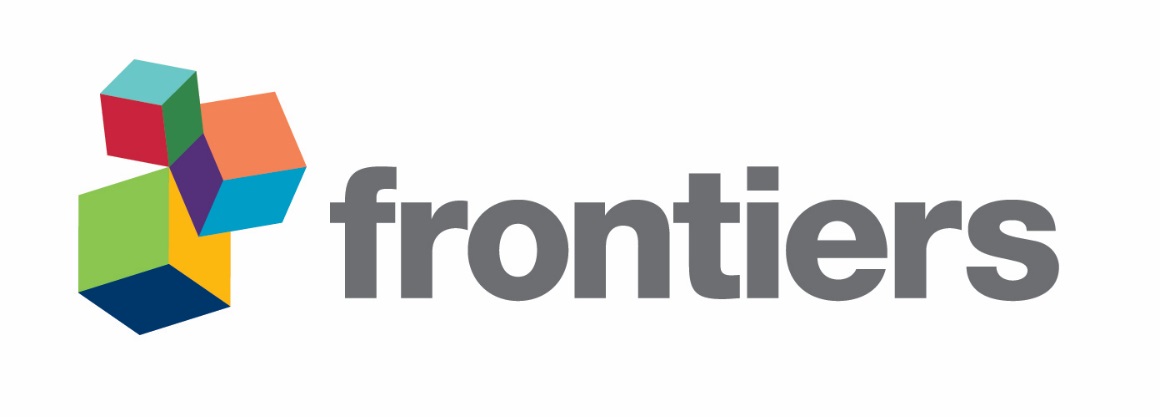
**

**Supplementary Figure 1.** Mendelian randomization analysis chart of AS and total CVD


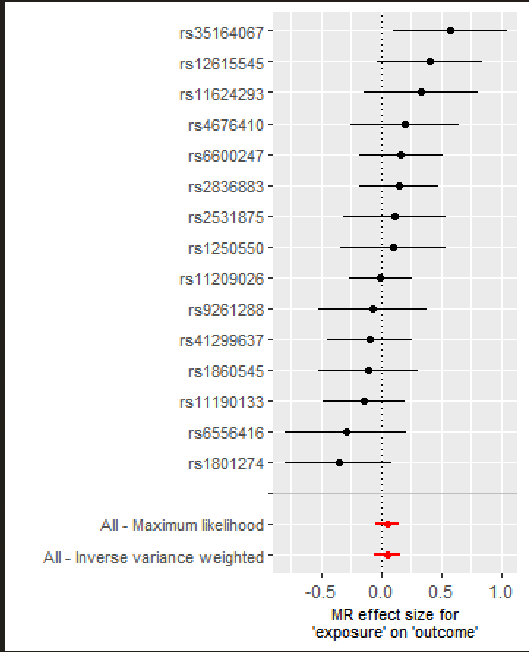

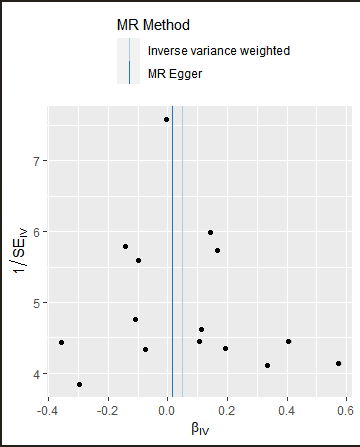

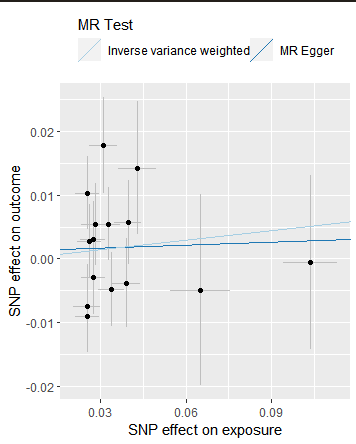

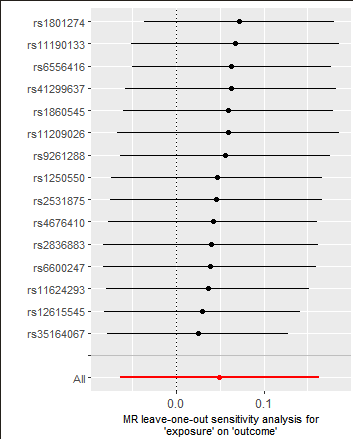


**Supplementary Figure 2.** Mendelian randomization analysis chart of AS and HF


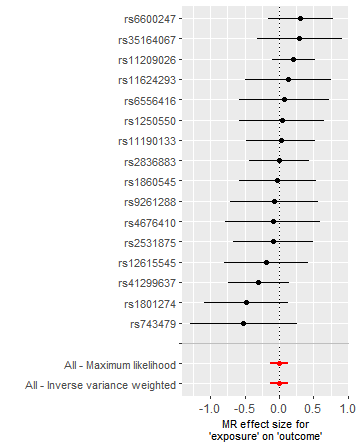

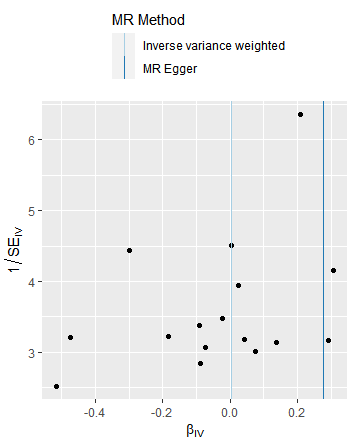

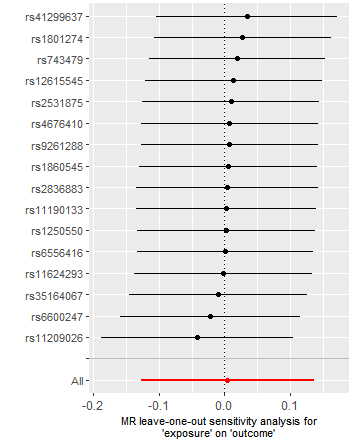

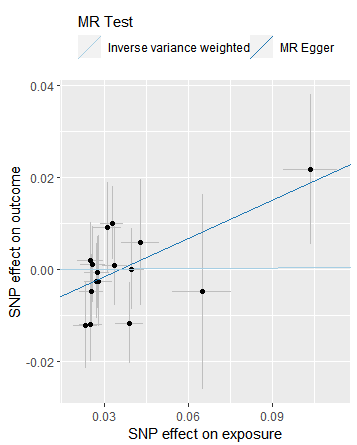


**Supplementary Figure 3.** Mendelian randomization analysis chart of AS and IHD


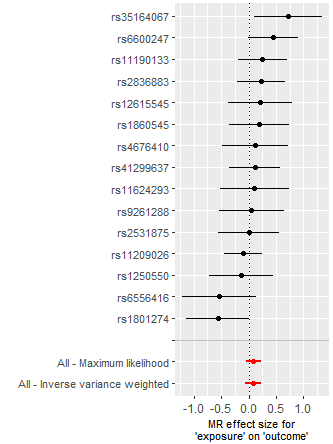

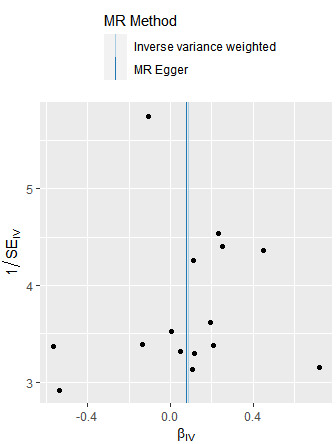

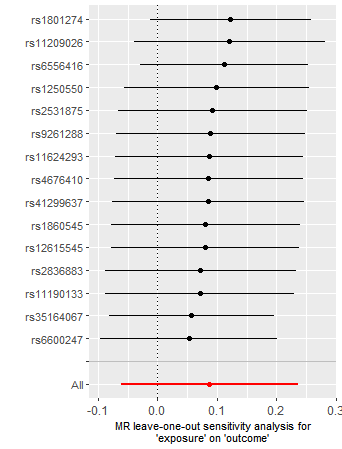

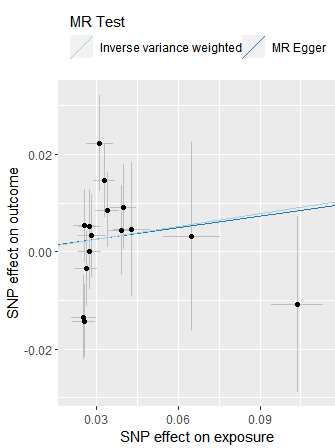


**Supplementary Figure 4.** Mendelian randomization analysis chart of AS and MI


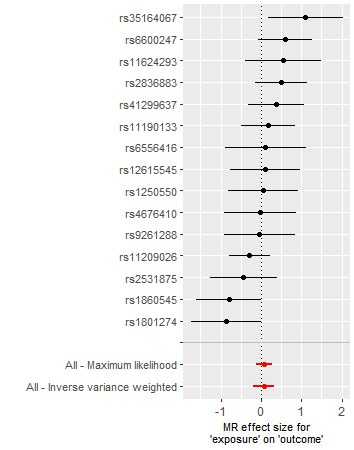

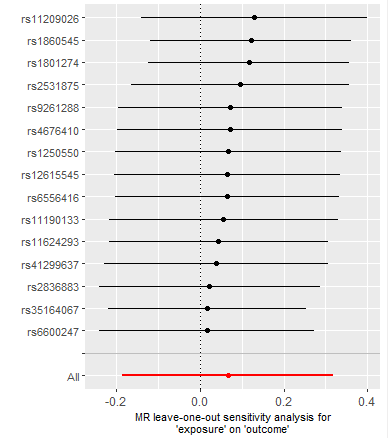

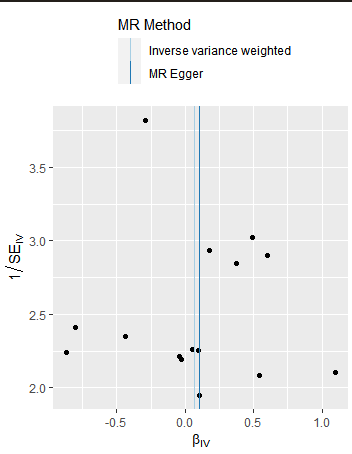

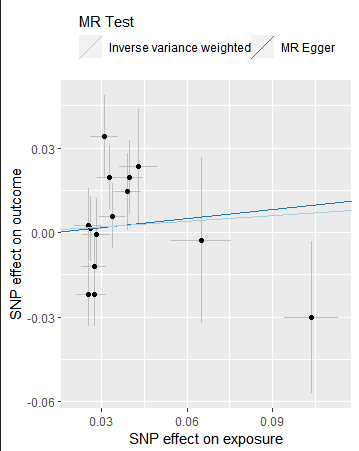


**Supplementary Figure 5.** Mendelian randomization analysis chart of AS and VHD


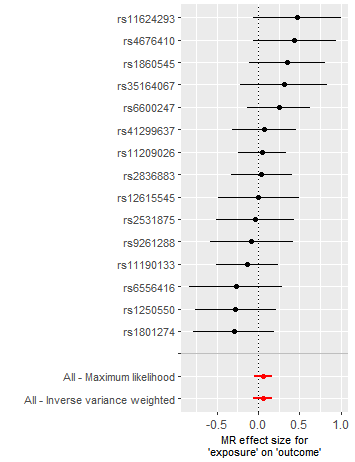

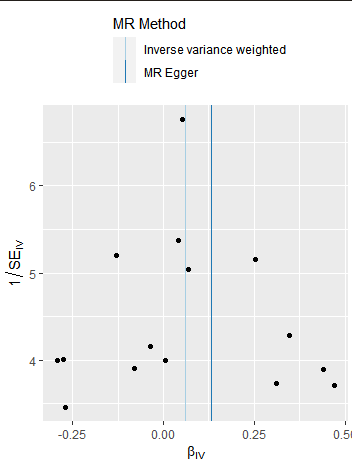

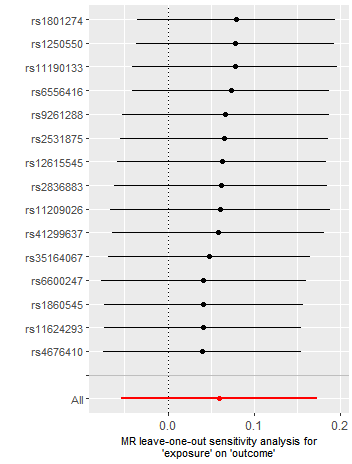

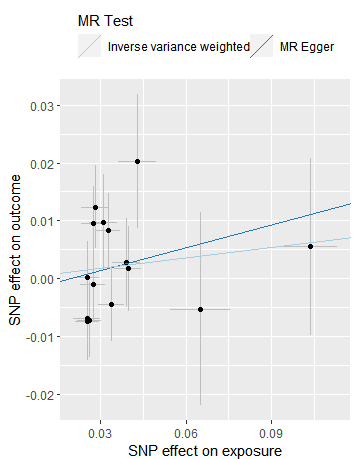


**Supplementary Figure 6.** Mendelian randomization analysis chart of AS and VTE


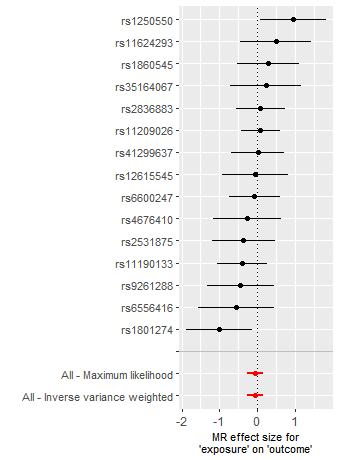

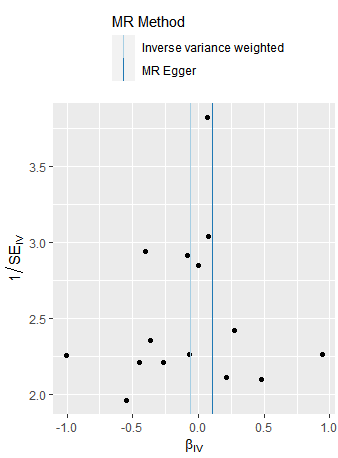

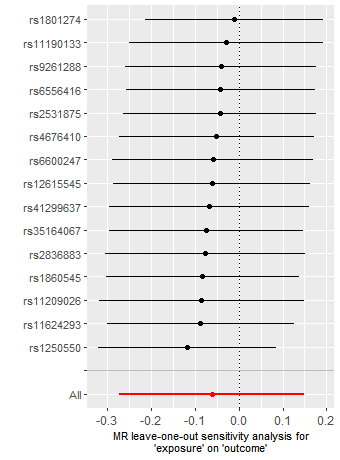

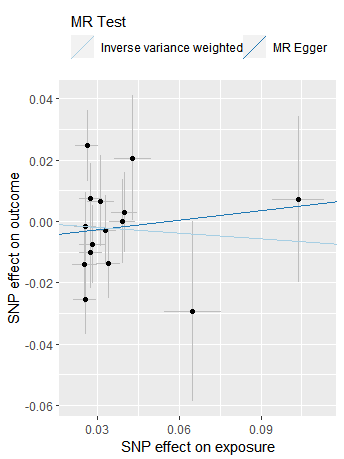


**Supplementary Figure 7.** Mendelian randomization analysis chart of AS and ASI


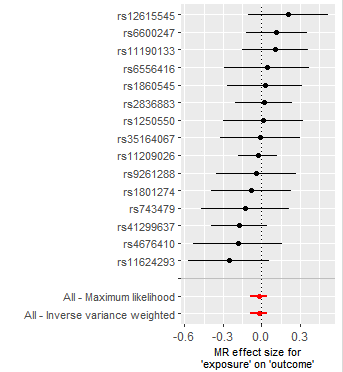

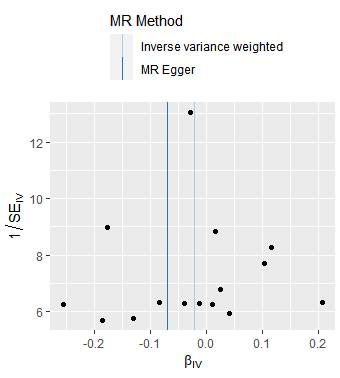

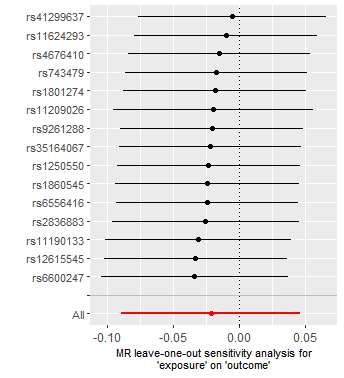

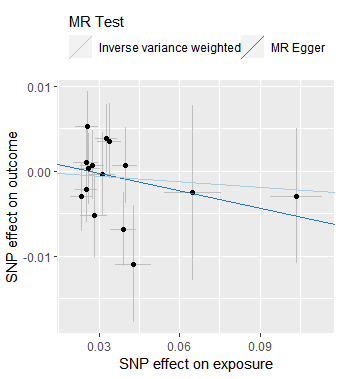


**Supplementary Figure 8** Mendelian randomization analysis chart of CVD and AS


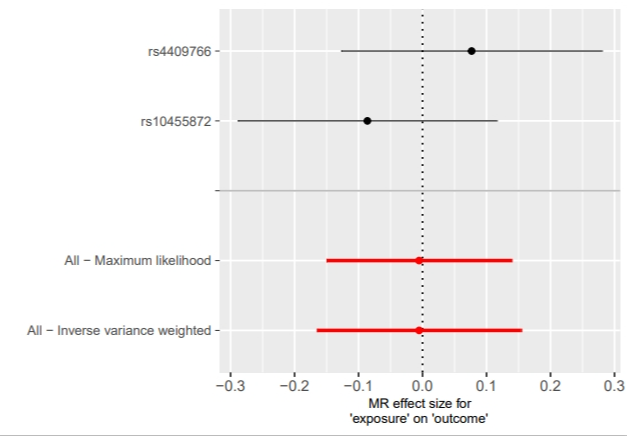

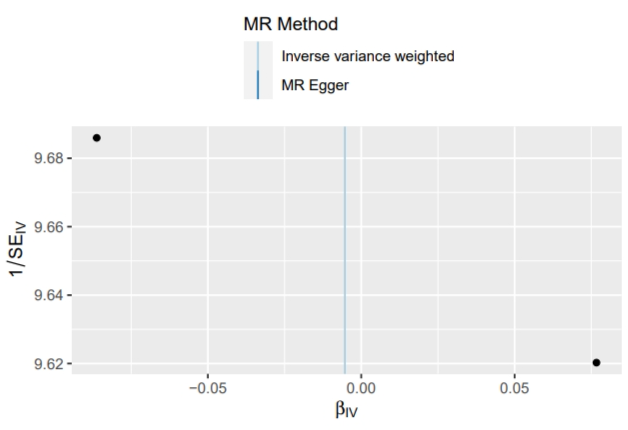


**Supplementary Figure 9** Mendelian randomization analysis chart of IHD and AS


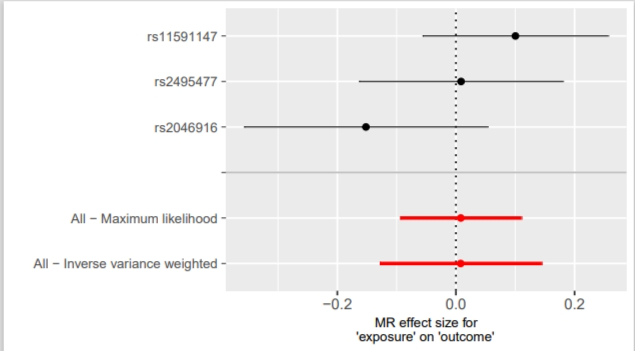

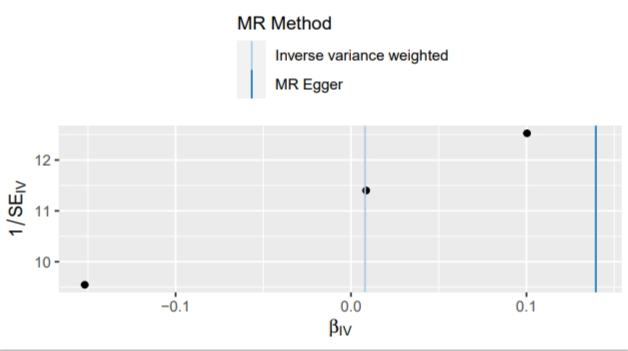

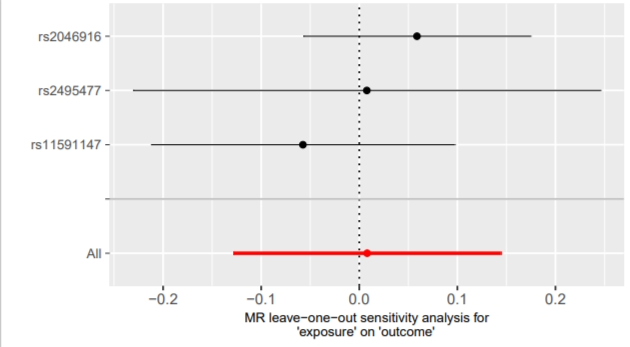


**Supplementary Figure 10** Mendelian randomization analysis chart of MI and AS


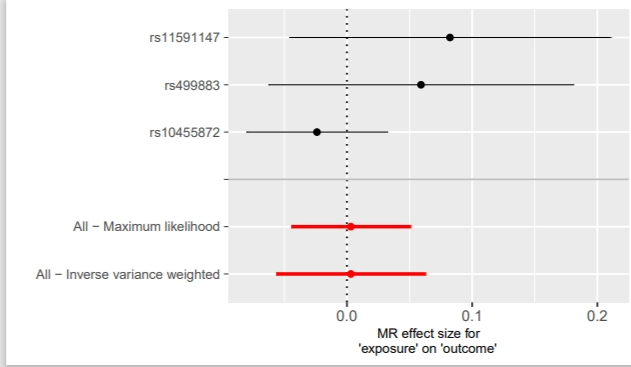

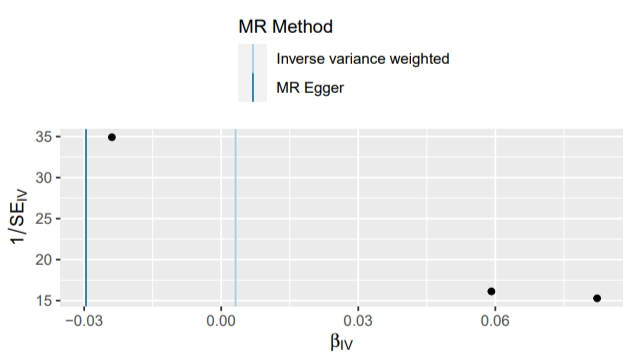

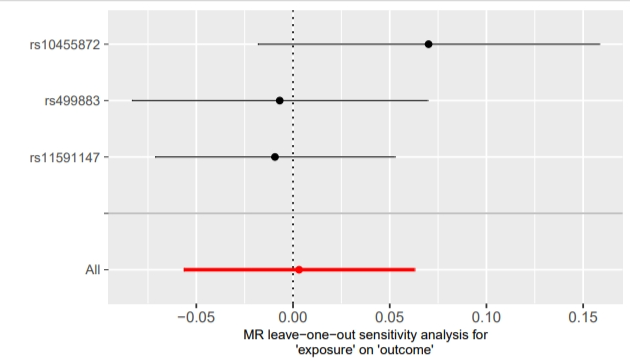

Supplement: Supplementary file 1 [file Table1.docx]
